# Reduced NAA-Levels in the NAWM of Patients with MS Is a Feature of Progression. A Study with Quantitative Magnetic Resonance Spectroscopy at 3 Tesla

**DOI:** 10.1371/journal.pone.0011625

**Published:** 2010-07-20

**Authors:** Fahmy Aboul-Enein, Martin Krššák, Romana Höftberger, Daniela Prayer, Wolfgang Kristoferitsch

**Affiliations:** 1 Department of Neurology, SMZ-Ost Donauspital, Vienna, Austria; 2 Department of Radiology, Medical University of Vienna, Vienna, Austria; 3 Institute of Neurology, Medical University of Vienna, Vienna, Austria; University of Texas M. D. Anderson Cancer Center, United States of America

## Abstract

**Background:**

Reduced N-acetyl-aspartate (NAA) levels in magnetic resonance spectroscopy (MRS) may visualize axonal damage even in the normal appearing white matter (NAWM). Demyelination and axonal degeneration are a hallmark in multiple sclerosis (MS).

**Objective:**

To define the extent of axonal degeneration in the NAWM in the remote from focal lesions in patients with relapsing-remitting (RRMS) and secondary progressive MS (SPMS).

**Material and Methods:**

37 patients with clinical definite MS (27 with RRMS, 10 with SPMS) and 8 controls were included. We used 2D ^1^H-MR-chemical shift imaging (TR = 1500ms, TE = 135ms, nominal resolution 1ccm) operating at 3Tesla to assess the metabolic pattern in the fronto–parietal NAWM. Ratios of NAA to creatine (Cr) and choline (Cho) and absolute concentrations of the metabolites in the NAWM were measured in each voxel matching exclusively white matter on the anatomical T2 weighted MR images.

**Results:**

No significant difference of absolute concentrations for NAA, Cr and Cho or metabolite ratios were found between RRMS and controls. In SPMS, the NAA/Cr ratio and absolute concentrations for NAA and Cr were significantly reduced compared to RRMS and to controls.

**Conclusions:**

In our study SPMS patients, but not RRMS patients were characterized by low NAA levels. Reduced NAA-levels in the NAWM of patients with MS is a feature of progression.

## Introduction

MS is a chronic inflammatory disease of the central nervous system (CNS) of unknown origin causing demyelination and axonal degeneration [1]. The immune reaction in MS patients is heterogeneous and compartmentalized within the CNS. It may be triggered temporarily or may take a continuous course [2]–[4].

The diagnosis of clinical definite MS is established by typical clinical symptoms, a relapsing remitting course and paraclinical findings in magnetic resonance imaging (MRI) and cerebrospinal fluid (CSF) [5]–[7]. Other disorders which may mimic MS must be ruled out.

In most cases MS follows a relapsing-remitting course (RRMS) with clearly defined relapses and no progression during clinical remissions [5]. It is unclear why MS sometimes follows a benign RRMS course while in other cases converting into a progressive-relapsing course, with or without superposed relapses (SPMS). It is further unknown, why MRI lesion load does not consistently correlate to clinical disability [5]–[8]. Supposedly even in very early stages of RRMS a “clinical silent” focal or diffuse “background inflammation” not detectable in conventional MRI may progress and cause diffuse axonal damage. This subtle axonal degeneration may be visualized by magnetic resonance spectroscopy (^1^H-MRS) as reduction of N-acetyl-aspartate (NAA) levels [9]–[14]. These results are, however, controversially discussed, due to new insights from the recently improved ^1^H-MRS technique. Higher field strengths (3 Tesla) and chemical shift imaging have substantially increased the validity of ^1^H-MRS. To our knowledge, so far only one study using multivoxel ^1^H-MR 2D spectroscopic imaging (MRSI) operating at 3 Tesla was published [15]. In this study, Kirov et al. found no reduction of NAA levels in 21 mildly disabled RRMS patients (mean expanded disability status scale (EDSS) of 1.4., mean disease duration of 2.3 years) compared to 15 matched controls.

The aim of our study was to define the axonal changes in the normal appearing white matter (NAWM) of SPMS and secondly, to reassess whether or not reduced NAA levels could be found in the NAWM of RRMS with improved MRSI operating at 3 Tesla.

## Materials and Methods

### Subjects

The study was approved by the local Ethics Committee (Commission of Medical Ethics of Vienna; Ethic Approval/Registration Number: EK 06-169-VK). Informed written consent was obtained from all patients and volunteers.

In total, 37 patients with clinically definite MS and characteristic MRI and CSF findings were included [6]. All patients showed oligoclonal bands. Twenty-seven patients followed a relapsing-remitting course with well defined relapses and lack of clinical progression between relapses. The RRMS patients were compared to 10 SPMS patients and 8 healthy controls (mean age, 46.3 years±6.3 [range, 17.0 to 65.0], female∶male = 7∶1). A complete survey of clinical data is given in table 1.

**Table 1:** Included MS patients.

patients	female∶male	age at onset ± STD (years)[range]	disease duration ± STD (months)[range]	number of relapses ± STD	EDSS at MRS
**RRMS**	**total, n = 27**	**30.2±2.2** **[13.0 to 56.0]**	**79.5±14.2** **[9.0 to 312.0]**	**3.9±0.4** **[2 to 9]**	**1.7±0.3** **[0.0 to 5.0]**
	female, n = 23	29.4±2.5[13.0 to 56.0]	88.1±15.8[14.0 to 312.0]	4.2±0.2[2 to 9]	1.8±0.3[0.0 to 5.0]
	male, n = 4	34.6±2.4[30.0 to 40.0]	30.0±16.8[9.0 to 80.0]	2.25±0.25[2 to 3]	1.1±0.7[0.0 to 3.0]
**SPMS**	**total, n = 10**	**27.25±9.2** **[13.0 to 41.5]**	**152.5±35.1** **[14.0 to 324.0]**	**5.5±0.9** **[2 to 10]**	**3.9±0.5** **[3.0 to 6.5]**
	female, n = 7	26.3±3.7[13.0 to 41.5]	144.0±46.4[14.0 to 324.0]	5.3±1.0[2 to 9]	3.4±0.2[3.0 to 4.5]
	male, n = 3	29.5±5.3[23.0 to 40.5]	172.3±55.5[78.0 to 278.0]	6.0±2.1[3 to 10]	5.3±1.2[3.0 to 6.5]

### MRI and MRS

We used the same standardized protocol for ^1^H-MR 2D Spectroscopic Imaging (MRSI) as described in detail previously [16].

MRSI at magnetic field strength of 3 Tesla (TimTrio equipped with standard head 8 channel receive coil, Siemens Healthcare, Erlangen, Germany) was applied to assess the metabolic pattern in the NAWM. MRSI measurements were performed parallel to axial T1- and T2-weighted slices. Routine automatic adjustments were applied prior to data acquisition. PRESS sequence was used for rectangular VOI selection (8×8×1 cm) in the fronto-parietal white matter well above lateral ventricles. Special care was taken to avoid skull and subcutaneous tissue contamination. CHESS sequence was used for water suppression. Sequence parameters included TR/TE 1500/135 ms, 16×16 elliptical weighted phase encoding steps across a 16×16 cm FOV, slice thickness of 1cm, 50% Hanning filter and 3 averages. To obtain intern quantification reference additional scan with the same timing and geometrical parameters was taken without water suppression. The acquisition time of spectroscopic sequences was less than 15 minutes.

Quantification of the spectra was performed off-line in the time domain using jMRUI software package [17]. Signals from voxels (8–12 voxels per RRMS patients and healthy controls, 3–8 voxels in SPMS patients) matching exclusively frontal and fronto-parietal white matter without any signs of lesion load on conventional T2 weighted images were selected. For the processing of metabolite spectra, remaining water signal was removed using HLSVD filter and amplitudes of Cho, Cr and NAA signals were calculated with AMARES [18]–[19]. Amplitude of water signal for each processed voxel was assessed from the scan without water suppression.

Concentrations of metabolites are given as ratios of signal intensities of NAA to Cr, NAA to Cho, and Cho to Cr respectively. Additionally, absolute concentrations of Cho, Cr and NAA were calculated using water signal from the identical voxel as internal reference. Mono exponential spin-lattice and spin-spin relaxation was assumed and published values of T1 (water: 832 ms; Cho: 1080 ms; Cr: 1240 ms; NAA: 1350 ms) and T2 (water: 110 ms; Cho: 187 ms; Cr: 156 ms; NAA: 295 ms) relaxation times of water and respective metabolites measured at 3T in NAWM of healthy volunteers were used for relaxation corrections [20]–[23]. The concentrations were calculated according to Kreis et al. as follows [24]:
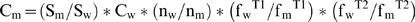
where C stands for concentration, S for signal intensity, n for the number of chemically equivalent protons, f^T1^ for spin-lattice relaxation function (1-e^TR/T1^), fT2 for spin-spin relaxation function (e^−TE/T2^) and the indexes m for metabolite and w for water. Cw was for white matter was chosen 35880 mmol/l according to Ernst et al. [25].

### Statistical analysis

Metabolic ratios and absolute concentrations obtained from individual voxels in NAWM were averaged for every patient. Intra-patient coefficient of variation averaged at 10% for Cho; 12% for Cr and 11% for NAA. Data are given as means for patient group. For statistical analysis nonparametric tests (Mann-Whitney, Kolmogorov-Smirnov) were applied (Statgraphics plus ©). All tests were classified as significant if the p-value was <0.05.

## Results

We studied 27 RRMS (mean disease duration, 5.6 years), 10 SPMS (mean disease duration, 12.7 years), and 8 healthy controls. Concentrations of metabolites were quantified in relation to each other as well as with respect to the concentration of internal tissue water of well defined regions of NAWM, and compared to recently published data [15].

**Figure 1:**
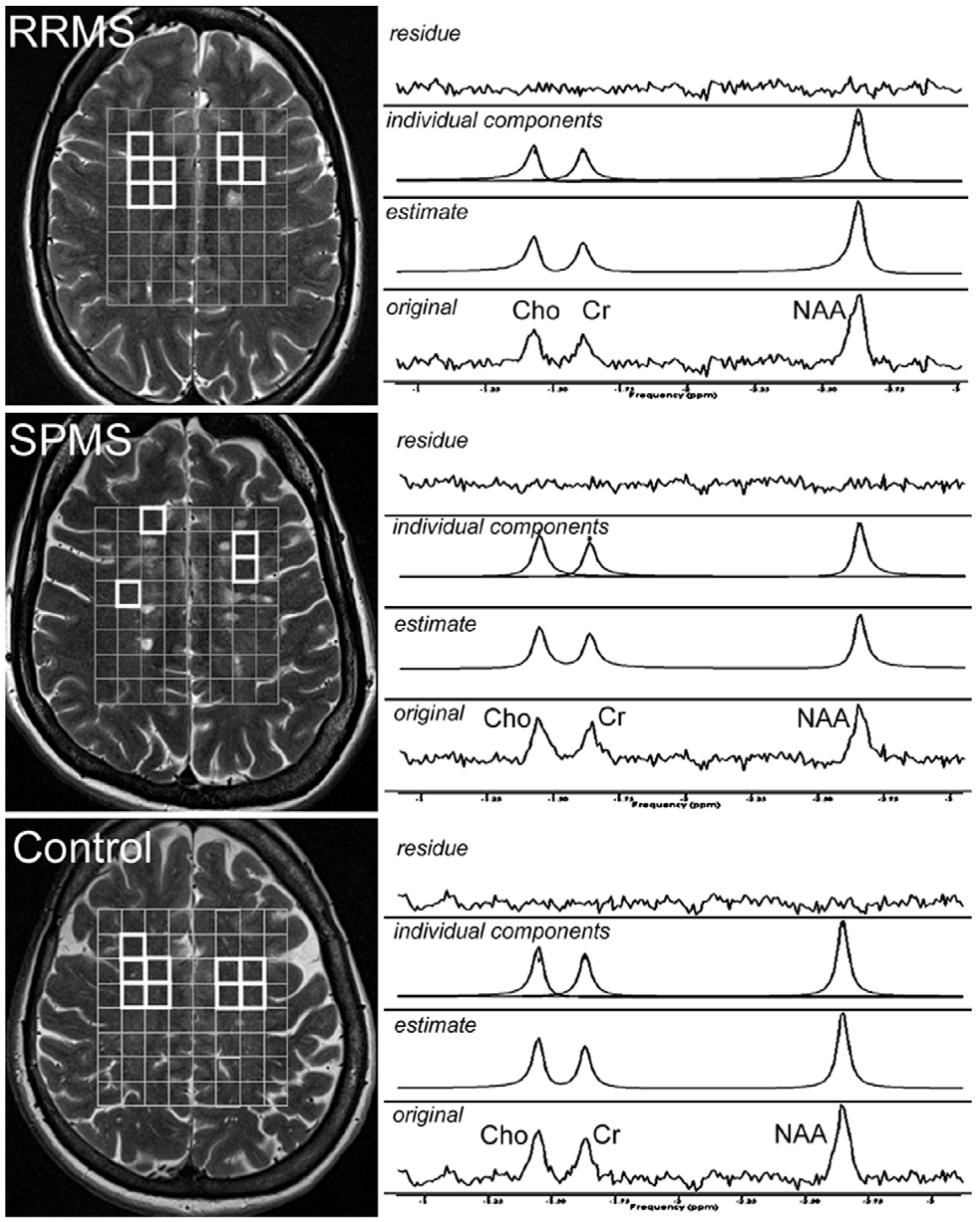
Spectral region including resonance lines of choline (Cho), creatine (Cr) and N-acetyl-aspartate (NAA). *Left column*, anatomical T2w MR Images with the depiction of MRSI PRESS box volume of interest (grey, 8×8 cm) and voxels (white) in the NAWM chosen for the spectroscopic data evaluation. *Right column*, representative MR spectra from a single voxel with original spectra (lower trace), AMARES estimate (second trace), individual components (third trace) and fit residue (upper trace). Data from RRMS (upper part) and SSPMS (middle part) patients as well as healthy controls (CON, lower part) are presented.

**Figure 2:**
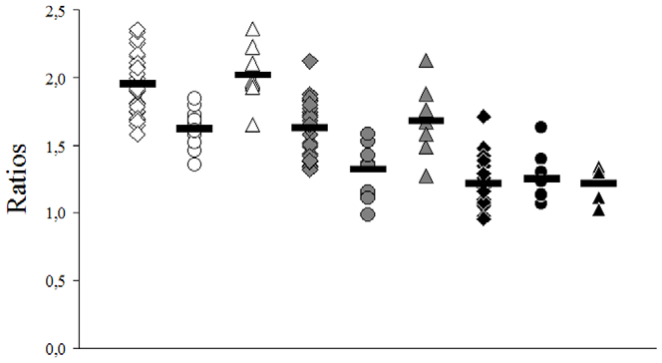
MRS Ratios of brain metabolite in NAWM of MS patients and controls. *white*, NAA/Cr ratio; *grey*, NAA/Cho ratios; black, Cho/Cr ratios; *squares*, RRMS patients (n = 27); *circles*, SPMS patients (n = 10); *triangles*, controls (n = 8); *bars*, means.

**Figure 3:**
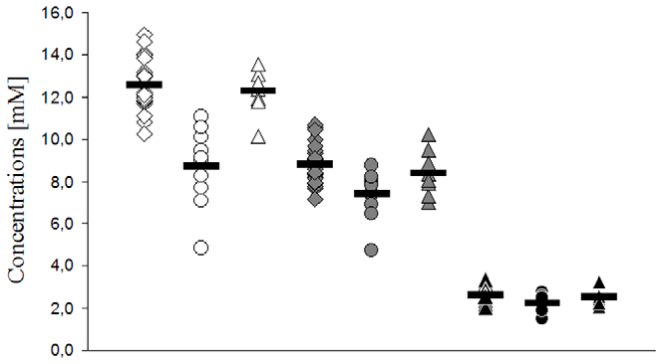
MRS absolute concentrations of brain metabolite in NAWM of MS patients and controls. *white*, NAA [mM]; *grey*, Cr [mM]; black, Cho [mM]. *squares*, RRMS patients (n = 27); *circles*, SPMS patients (n = 10); *triangles*, controls (n = 8); *bars*, means.

Examples of MR spectra of RRMS, SPMS and healthy controls are given in figure 1. Relative standard deviation of spectral fitting for all included spectra was ≤5%. All MRS data of the NAWM are presented in detail in figure 2 (ratios of metabolites, relative concentrations) and in figure 3 (absolute metabolic concentrations). Our results may be summarized briefly as follows.

### 1. Metabolite ratios, NAA/Cr, NAA/Cho and Cho/Cr

We found significant differences of metabolite ratios of RRMS (n = 27) compared to SPMS (n = 10) (NAA/Cr, p<0.0003; NAA/Cho, p<0.001), and between SPMS and controls (n = 8) (NAA/Cr, p<0.001; NAA/Cho, p<0.008).

No significant differences could be detected in all other comparisons (RRMS vs. controls, NAA/Cr, NAA/Cho and Cho/Cr and SPMS vs. controls, Cho/Cr) (figure 2).

### 2. Absolute metabolite concentrations [mM], NAA, Cr and Cho

#### NAA (figure 3, white symbols)

No significant difference of absolute NAA concentrations was found between RRMS (n = 27; NAA [mM] mean = 12.4±0.5; range 10.1 to 14.7) and controls (n = 8; NAA [mM] mean = 12.3±0.4; range 10.1 to 13.6). In SPMS NAA-levels (n = 10; NAA [mM] mean = 8.6±0.7; range 4.9 to 11.1) were significantly reduced compared to RRMS (n = 27; p<0.00001) and to controls (n = 8; p<0.001).

#### Cr (figure 3, grey symbols)

Absolute concentrations of Cr were found significantly reduced in SPMS (n = 10; Cr [mM] mean = 7.4±0.4; range 4.7 to 8.8) compared to RRMS (n = 27; Cr [mM] mean = 8.8±0.2; range 7.2 to 10.2) (p<0.0008). No significant differences were found between SPMS and controls (n = 8; Cr [mM] mean = 8.4±0.4; range 7.0 to 10.2), and between RRMS patients and controls.

#### Cho (figure 3, black symbols)

Absolute concentrations of Cho differed slightly between RRMS (n = 27; Cho [mM] mean = 2.6±0.1; range 2.1 to 3.3) and SPMS (n = 10; Cho [mM] mean = 2.2±0.1; range 1.5 to 2.8) (p<0.2). No differences of Cho concentrations were found between RRMS and controls (Cho [mM] mean = 2.5±0.1; range 2.1 to 3.2), and SPMS and controls.

#### Relation of absolute metabolite concentrations and EDSS

We found only a weak, but significant correlation between reduced NAA levels and higher EDSS (linear regression: correlation coefficient −0.48, R^2^ = 23.4, standard error for the estimate = 1.9, ANOVA table p<0.002). No correlations were found between Cr or Cho concentrations and EDSS.

In summary, we found a significant reduction of NAA/Cr and NAA/Cho ratios and of absolute concentrations of NAA and Cr in SPMS patients only. Between RRMS and controls, ratios and absolute concentrations for the examined metabolites did not show significant differences.

## Discussion

In our study we analyzed NAA, Cr and Cho levels of the NAWM of 37 MS patients and controls with multivoxel ^1^H-MRSI operating at 3 Tesla. Our results for 27 RRMS patients and 8 controls are in accordance with recently published data of 21 RRMS and 15 controls [15]. In addition we found significant changes in the NAWM of 10 SPMS.

^1^H-MRS allows an assessment of subtle metabolic changes within the NAWM [12], [14]. NAA is almost exclusively found in active mitochondria of neurons and axons and is thus a marker of axonal function and integrity [26]. Reduced levels of NAA are suggestive for axonal degeneration or axonal loss, or both [26]. They may correlate to histopathological changes and proposed histotoxic mechanisms in MS [4], [27]. Whether reduced NAA levels in MS patients reflect microscopic axonal changes due to primary tissue damage, or due to axonal transection in remote demyelinating lesions remains unclear [28]. While some studies found decreased NAA levels in the NAWM of MS patients [28]–[29], others did not [13], [15], [30]. These divergent results may be explained by the very small and heterogeneous patient cohorts (e.g. 2 RRMS and 12 SPMS aged from 28 to 61 years, and 4 controls aged 33 to 48 years in van Walderveen *et al.*, 1999 [29]), by the use of a 1.5 Tesla ^1^H-MRS and by the measurement of metabolite ratios instead of absolute metabolite concentrations [30]–[32].

The signal-to-noise ratio and spectral resolution are markedly reduced in magnetic field strengths lower than 3 Tesla. This suggests the use of ^1^H-MRS operating at 3 Tesla instead of 1.5 Tesla [11], [32]–[33]. Furthermore only the improved MRSI allows for a robust quantification of the respective signals simultaneously in large brain regions [11], [32]–[33].

Metabolite ratios are less sensitive than absolute concentrations of individual metabolites [33], [35]. The quantification of the metabolite concentration is usually based on the metabolite signal ratio to Cr which is regarded as “internal reference” for metabolite ratios [34]–[35]. But the concentration of Cr may vary in different pathological conditions and at different age [32], [34]–[35]. The Cr peak is composed of creatine and phosphocreatine. Cr occurs mainly in glial cells, and is considered as a marker of cell energy metabolism and mitochondrial function [34]. Increased Cr levels may indicate glial cell proliferation or higher glial cell metabolism and tissue repair mechanisms [32]. NAA levels might be normal, because neuronal cell bodies remote from the focal or diffuse white matter lesions may be unaffected and may maintain the axonal transport of mitochondria to their distal ends. Markedly reduced Cr levels might reflect the complete collapse of glial cell metabolism preceding axonal changes, significant reduction of glial cells, or tissue scar formation.

Lower NAA/Cr ratios must therefore not per se be interpreted as axonal dysfunction or loss, if absolute NAA levels remain unchanged but Cr levels are increased [31], [34]–[37]. Vrenken et al., for example, found significantly reduced NAA/Cr ratios in the NAWM of 76 MS patients (RRMS, SPMS and PPMS), while absolute concentrations for NAA were not reduced but Cr was increased [31]. They concluded that increased glial cell numbers, and not axonal loss or dysfunction might be a common feature in the NAWM of MS patients [31].

In conclusion, we want to emphasize that the normal NAA levels found in our RRMS patients do not necessarily indicate normal NAWM. Tissue changes in MS patients are heterogeneous and too complex to be detected by measuring only few metabolites [4]. Spectral appearance and spin-spin relaxation properties of further metabolites that might be changing in the NAWM (e.g. glutamate, glutamine, myo-Inositol and lipids) favour the measurements with shorter echo time, whereas flatter spectral baseline at longer echo times increases the precision of the NAA, Cr and Cho measurements.

MRSI was useful for demonstrating changes in the NAWM of SPMS in our and in other studies clearly [13], [29]. But there is still an ongoing discussion, whether MRSI is sensitive to detect also subtle axonal changes in the NAWM of benign or stable RRMS patients. However, reduced NAA levels in RRMS without signs of progression might be a strong indicator for clinically still undetectable progressive axonal degeneration. This could identify patients with progressive RRMS at a very early stage prior to clinical deterioration [3]–[4]. MRS therefore may provide crucial information for treatment decision, as lesion load does not necessarily correlate with disease activity and progression [1], [3], [8], [38].
